# Tubular *IKKβ* Deletion Alleviates Acute Ischemic Kidney Injury and Facilitates Tissue Regeneration

**DOI:** 10.3390/ijms231710199

**Published:** 2022-09-05

**Authors:** Eileen Dahlke, Toni Engmann, Yaman Anan, Robert Häsler, Giovanni Solinas, Franziska Theilig

**Affiliations:** 1 Institute of Anatomy, Christian Albrechts-University Kiel, 24118 Kiel, Germany; 2 Department of Dermatology and Allergy, University Hospital Schleswig-Holstein, 24105 Kiel, Germany; 3 Department of Molecular and Clinical Medicine, University of Göteborg, 405 30 Göteborg, Sweden; 4 Institute of Anatomy, Department of Medicine, University of Fribourg, 1700 Fribourg, Switzerland

**Keywords:** IKKβ, AKI, NF-ĸB, tissue regeneration

## Abstract

Acute kidney injury (AKI) is a common renal injury leading to relevant morbidity and mortality worldwide. Most of the clinical cases of AKI are caused by ischemia reperfusion (I/R) injury with renal ischemia injury followed by reperfusion injury and activation of the innate immune response converging to NF-ĸB pathway induction. Despite the clear role of NF-ĸB in inflammation, it has recently been acknowledged that NF-ĸB may impact other cell functions. To identify NF-ĸB function with respect to metabolism, vascular function and oxidative stress after I/R injury and to decipher in detail the underlying mechanism, we generated a transgenic mouse model with targeted deletion of IKKβ along the tubule and applied I/R injury followed by its analysis after 2 and 14 days after I/R injury. Tubular IKKβ deletion ameliorated renal function and reduced tissue damage. RNAseq data together with immunohistochemical, biochemical and morphometric analysis demonstrated an ameliorated vascular organization and mRNA expression profile for increased angiogenesis in mice with tubular IKKβ deletion at 2 days after I/R injury. RNAseq and protein analysis indicate an ameliorated metabolism, oxidative species handling and timely-adapted cell proliferation and apoptosis as well as reduced fibrosis in mice with tubular IKKβ deletion at 14 days after I/R injury. In conclusion, mice with tubular IKKβ deletion upon I/R injury display improved renal function and reduced tissue damage and fibrosis in association with improved vascularization, metabolism, reactive species disposal and fine-tuned cell proliferation.

## 1. Introduction

Acute kidney injury (AKI) is a common renal injury leading to relevant morbidity and mortality, causing growing economic burden to the health care systems [1]. AKI may have different causes, classically classified as prerenal (decreased perfusion), renal (parenchyma cell injury) and post-renal (urinary tract obstruction) origin. Most of the clinical cases of AKI are caused by ischemia reperfusion (I/R) injury due to renal hypoperfusion after surgery, hemorrhage, cardiac shock or sepsis [2]. The first event is the interruption of blood supply with deprivation of oxygen and nutrients triggering cell injury and cell death. This is followed by the reperfusion injury with generation of reactive oxygen species (ROS), mitochondrial failure, endothelial dysfunction and sterile inflammation [3]. The innate immune response is activated after reperfusion and signal transduction pathways converge on NF-ĸB. Kidney injury relevant stimuli include cytokines, growth factors, pathogen-associated damage and metabolic stress activating the IĸB kinase (IKK) complex, composed of IKKα, IKKβ and IKKγ. The IKK complex is responsible for the activation of NF-ĸB/Rel family of transcription factors through direct phosphorylation by IKKβ of the inhibitor protein IĸBα, leading to IĸBα ubiquitination and proteolytic degradation. The resulting nuclear translocation of the canonical NF-ĸB members (p65-p50 dimers) stimulate the transcription of proinflammatory genes and many chemokines, creating a positive feedback loop [2]. Several approaches of targeting NF-ĸB in I/R injury have been demonstrated beneficial when NF-ĸB is inhibited in the early phase of inflammation after I/R injury [2]. Using transgenic mouse models with tubule-specific expression of an IĸBα mutant that cannot be phosphorylated by IKKβ after I/R injury demonstrated improved renal function and reduced expression of NF-ĸB-dependent genes [4]. By contrast, lymphocyte-specific deletion of IKKβ worsened kidney function after I/R injury [5] demonstrating the importance of cell-type specific intervention. Although renal function was improved in tubule-specific prevention of NF-ĸB activation after I/R injury, the underlying mechanisms are still incompletely understood. Additionally, the long-term outcome and kidney repair mechanisms remain elusive. Despite the clear role of NF-ĸB in inflammation and tissue injury after I/R, it has recently been acknowledged that NF-ĸB may exert a broader impact [6].

To decipher in detail the underlying mechanism of how a prevention of tubular NF-ĸB-mediated inflammatory response to I/R injury ameliorates renal function and possibly tissue regeneration, we have generated an inducible transgenic mouse model with targeted deletion of *IKKβ* along the tubule and applied I/R injury. We can demonstrate that preventing tubular NF-ĸB-mediated response ameliorates vascular function, cell metabolism and oxidative stress handling after I/R injury leading to an overall improved recovery at two weeks after I/R injury.

## 2. Results

### 2.1. Genetic Tubular IKKβ-Deletion Ameliorates I/R Injury and Reinforces Tissue Regeneration

**Figure 1:**
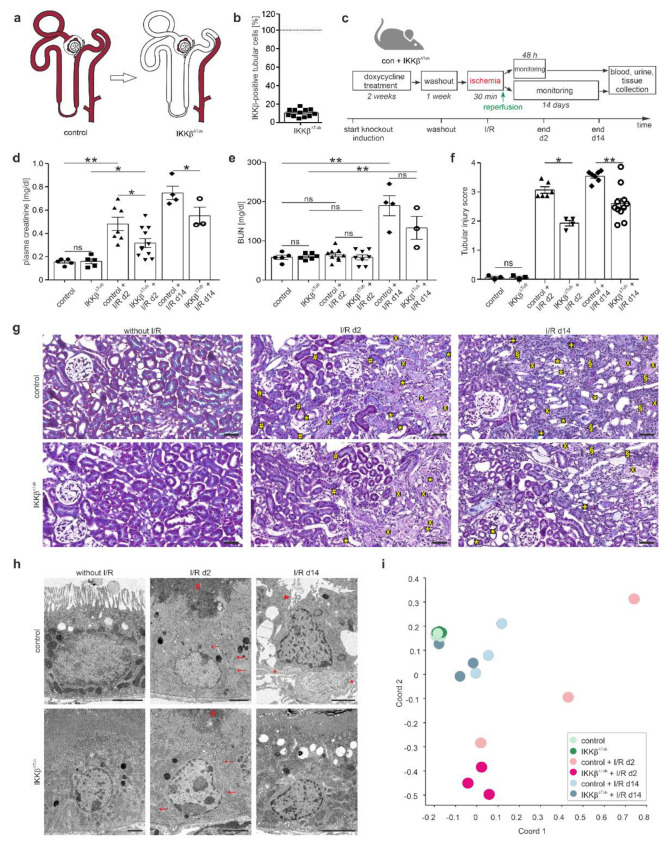
Genetic tubular IKKβ-deletion ameliorates I/R injury. (**a**) Scheme illustrating deletion of *IKKβ* gene under Pax8 promoter along the tubule; (**b**) semi-quantitative analysis of IKKβ mRNA expression derived from Exon 3 in tubular cells of control and IKKβ^∆Tub^. Arithmetic means ± SEM of *n* = 12 per group; * *p* < 0.05, *t*-test; (**c**) timeline of experimental procedure; (**d**,**e**) plasma creatinine (**d**) and blood urea nitrogen (BUN; (**e**)) values of control and IKKβ^∆Tub^ at baseline, 2 days and 14 days after I/R injury. Arithmetic means ± SEM of *n* = 3–11 per group; (**f**) semi-quantitative evaluation of tubulointerstitial injury. Arithmetic means ± SEM of *n* = 3–11 per group; (**g**) representative images of Masson trichrome stained kidney section of control and IKKβ^∆Tub^ at baseline, 2 days and 14 days after I/R injury. Scale bar = 50 µm. Tubular necrosis (x), loss of brush border (*), eosinophilic debris (#) and cellular infiltration (§) and fibrotic areas (+) are shown; (**h**) representative electron microscopy images of control and IKKβ^∆Tub^ at baseline, 2 days and 14 days after I/R injury. Scale bar = 2 µm. Reduced visibility of mitochondria (arrow), cellular debris (#), loss of brush border (arrow head) and basement membrane thickening (*) are encountered; (**i**) principal coordinate analyses between control and IKKβ^∆Tub^ at baseline, 2 days and 14 days after I/R injury displayed as variation between samples; IKKβ^∆Tub^ vs. control at baseline, at 2 days and 14 days after I/R injury based on filter criteria DESeq *p*-values < 0.05; fold change > 1.5. For (**d**–**f**) * *p* < 0.05, ** *p* < 0.01, Mann–Whitney–U test (if *n* < 4 Lord test).

To examine the effects of the involvement of tubular NF-ĸB activation in renal damage mechanism and in tissue regeneration after I/R injury, we generated mice with deletion of *IKKβ* along the tubule by cross-breeding IKKβ^flox^ mice, termed control, with Pax8rt-TA-LC1^Cre^ mice, termed IKKβ^∆Tub^ (Figure 1a). Determining the degree of *IKKβ* knockout by quantifying the *IKKβ*-Exon 3 derived mRNA expression in tubular cells revealed an 89.4 ± 4.2% deletion of *IKKβ* along the tubule in IKKβ^∆Tub^ (Figure 1b). After knockout induction and wash out, I/R injury was induced and monitored for either 48 h (2 d) and 14 days (14 d) in control and IKKβ^∆Tub^ mice (Figure 1c). Renal function was assessed. Plasma creatinine levels were significantly higher after I/R injury at 48 hours and remained high after 14 days (Figure 1d). Deletion of *IKKβ* alleviated the high plasma creatinine levels after I/R injury in IKK^β∆Tub^ mice at 2 and 14 days after I/R injury compared to their respective control mice. Blood urea nitrogen (BUN) was not different between strains at baseline nor at 2 days after I/R injury (Figure 1e). At 14 days after I/R injury, BUN levels were significantly higher in both strains and were reduced in IKKβ^∆Tub^ compared to their respective control, although not significantly due to the low n-number. Histological assessment of the degree of tubule-interstitial damage using an established scoring system demonstrated more intense damage in control mice at 2 and 14 days after I/R compared to IKKβ^∆Tub^ mice (Figure 1f). These histological analyses indicated an amelioration of cortical and outer stripe tubular ischemic injury in IKKβ^∆Tub^ mice as they presented fewer less loss of brush border, accumulation of luminal cellular debris, infiltrating cells and fibrotic areas at 2 and 14 days after I/R injury (Figure 1g). Electron microscopy analysis of proximal tubule cells from the S1 and S2 segments revealed at 2 days after I/R injury in IKKβ^∆Tub^ mice less mitochondrial swelling and luminal accumulation of cellular debris and less nuclear damage compared to control (Figure 1h). At 14 days after I/R injury in comparison to control, IKKβ^∆Tub^ mice presented less loss of brush border membrane and basement membrane thickening. To determine underlying mechanisms, RNA sequencing was performed. Principle coordinate analyses between strains and I/R injury displayed as variation between samples (for processed data analysis see Supplemental Table S1). As illustrated in Figure 1i, control and IKKβ^∆Tub^ mice at baseline did not vary much between strains. At 2 days after I/R injury changes, sample clustering was induced, which differed between strains and from baseline. At 14 days after I/R injury, sample clustering moved to control levels and strain difference was reduced. Note that sample clustering of IKKβ^∆Tub^ mice at 14 days after I/R injury was closer to baseline levels as compared to control after I/R injury.

### 2.2. Comparison of Gene Expression Levels of IKKβ^∆Tub^ Mice Compared to Control at Baseline

**Figure 2:**
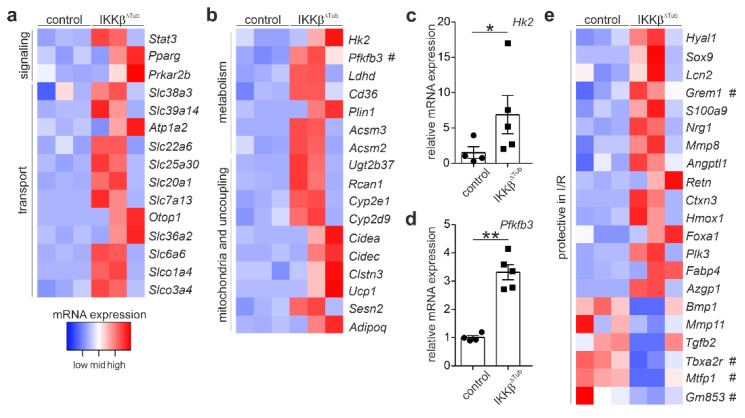
Tubular deletion of IKKβ alters baseline gene expression. (**a**) heatmaps of significantly increased mRNA of proteins important in signaling transduction and epithelial transport; (**b**) Heatmaps of significantly augmented mRNA expression levels coding for enzymes of metabolism (glycolysis, of lipid uptake, storage and biosynthesis) and mitochondria and mitochondrial uncoupling. IKKβ^∆Tub^ vs. control based on genes, selected by the filter criteria DESeq *p*-values < 0.05; fold change > 1.5; (**c**,**d**) real-time PCR of Hk2 and Pfkfb3. Arithmetic means ± SEM of *n* = 4–5 per group; * *p* < 0.05, ** *p* < 0.01 using Lord test; (**e**) heatmaps of significantly altered genes known to ameliorate the extent of I/R injury. (#) marks the most significantly differentially altered mRNA expression level. All heatmaps show samples organized in columns and genes in rows, expression intensities are color-coded.

To identify which gene alterations of IKKβ^∆Tub^ compared to control altered before the induction of I/R injury could attenuate the extent of I/R injury, we compared gene expression of both strains at baseline. Among the significantly altered genes, we identified genes coding for cellular signal transduction, various membrane transporter, enzymes affecting cell metabolism and mitochondria function including mitochondria uncoupling; and genes coding for proteins whose alterations were shown to ameliorate I/R injury (Figure 2). We observed and strong augmentation of Pfkfb3 mRNA coding for 6-phosphofructo-2-kinase/fructose-2,6-biphosphatase isoform 3 (PFKFB3), a key enzyme for glycolysis in IKKβ^∆Tub^ compared to control (Figure 2b). In congruent with augmented glycolysis, we found a significant increased expression of Hk2 and Ldhd mRNA, coding for hexokinase 2 (HK2) and lactate dehydrogenase D, respectively. The expression level of Hk2 and Pfbkb3 was confirmed by real-time PCR (Figure 2c,d) showing significant increased mRNA levels for HK2 and PFKFB3 in IKKβ^∆Tub^ compared to control. Additionally, lipid metabolism was also affected. We identified Cd36, coding for CD36 and important for cellular lipid uptake; Plin1, coding for perlipin 1 and important for lipid storage and lipid droplet expansion; and Acsm2 and Acsm3, coding acyl CoA-synthetase 2 and 3 important for fatty acid biosynthesis. Furthermore, among the genes which were shown to ameliorate the extent of I/R injury, most significantly different expressed were Grem1, coding for gremlin-1, Tbxa2r, coding for thromboxane A2 receptor (TBXA2R) and Gm853, coding for leucine decarboxylase (Figure 2e). For example, gremlin-1 was shown to antagonize TGFβ function and to reduce infarct size after myocardial ischemia [7]. Additionally, using salt loaded stroke-prone spontaneously hypertensive rats, pharmacological inhibition of TBXA2R was shown to reduce oxidative stress, Hypoxia-inducible factor-1 expression and renal glomerular and tubulointerstitial damage [8].

### 2.3. Tubular Deletion of IKKβ Promotes Angiogenesis after I/R Injury and Preserves Tubulointerstitial Architecture

**Figure 3:**
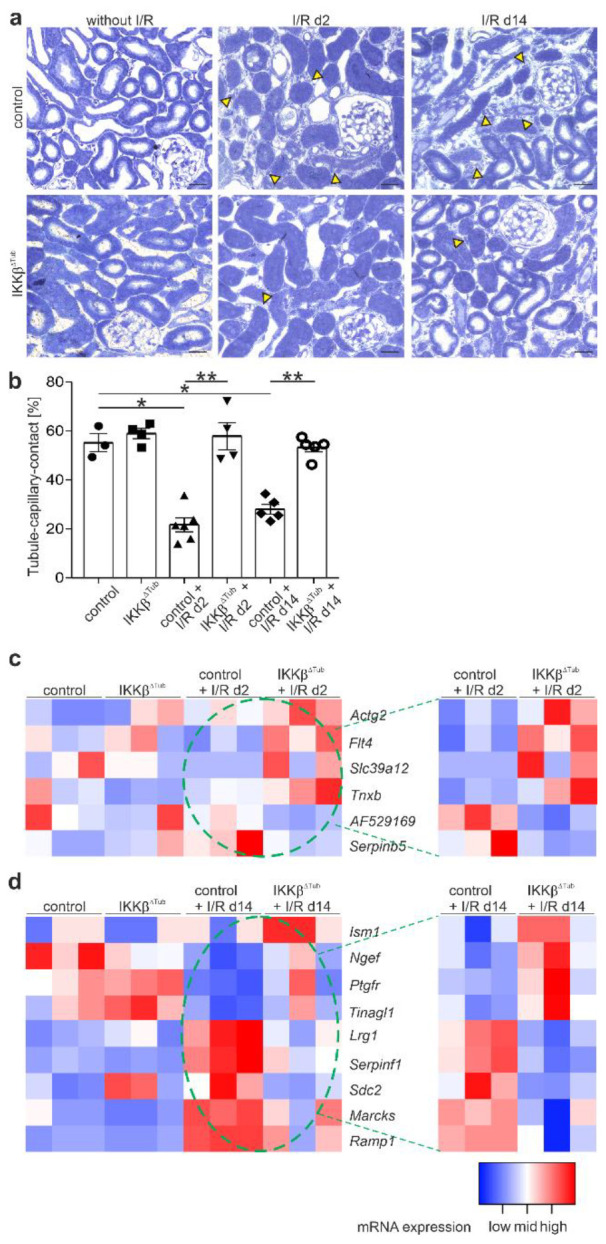
Tubular deletion of IKKβ induces angiogenesis. (**a**) representative images of semi-thin sections from control and IKKβ^∆Tub^ at baseline, 2 days and 14 days after I/R injury. Scale bar = 50 µm; (**b**) morphometric analysis of tubule–capillary contact length in %. Arithmetic means ± SEM of *n* = 3–6 per group; * *p* < 0.05, ** *p* < 0.01 using a Mann–Whitney–U test (if *n* < 4 Lord test). (**c**,**d**) Heatmaps of significantly altered mRNA of proteins known to affect angiogenesis at 2 days after I/R injury compared to baseline (**c**) and at 14 days after I/R injury compared to baseline (**d**). Filter criteria were DESeq *p*-values < 0.05; fold change > 1.5. All heatmaps show samples organized in columns and genes in rows, expression intensities are color-coded. The green stippled circle indicates mRNA of I/R groups used for relative expression in heatmaps by comparing only control with IKKβ^∆Tub^ at 2 days (**c**) and 14 days (**d**) after I/R injury.

In animal models with acute kidney injury, impairment of renal blood flow was shown to be associated with diminished renal function and tissue damage. Paracrine factors from stressed epithelium represented a potential link between epithelial and vascular compartment [9]. We therefore performed a morphometric analysis of the microvasculature of renal cortices and outer stripes by measuring the tubule–capillary contact area. At baseline, tubule–capillary contact length in IKKβ^∆Tub^ did not differ from control (Figure 3a,b). At 2 and 14 days after I/R injury, tubule–capillary contact length remained normal in IKKβ^∆Tub^ and was significantly reduced in control. To identify the underlying mechanism, we analyzed altered mRNA expression levels from RNA Seq results for significantly altered mRNA involved in angiogenesis. Altered mRNA involved in angiogenesis differed between at days 2 and 14 after I/R injury (Figure 3c,d). At day 2 after I/R injury in IKKβ^∆Tub^ compared to control, we found significantly augmented mRNA levels of Actg2 (smooth muscle actin γ2), Flt4 (vascular endothelial growth factor receptor 3), Slc39a12 (zinc transporter 12) and Tnxb (tenascin XB), which are known to promote angiogenesis and angiogenic sprouting among others by inducing proliferation and migration of endothelial and smooth muscle cells. Furthermore, at day 2 after I/R injury in IKKβ^∆Tub^ compared to control, reduced mRNA levels were found for Minar1 (major intrinsically disordered Notch2-binding receptor 1, AF529169) and Sepinb5 (serpin family B member 5), both are known angiogenesis inhibitors. At 14 days after I/R injury in IKKβ^∆Tub^ compared to control, we found significantly augmented mRNA levels of Ism1 (isthmin1), Ngef (neuronal guanine nucleotide exchange factor), Ptgfr (prostaglandin F receptor), and Tinagl1 (tubulointerstitial nephritis antigen like 1). Whereas Ism1 is an inhibitor for angiogenesis, Ngef, Ptgfr and Tinagl1 are pro-angiogenic factors. However, mRNA levels significantly reduced in IKKβ^∆Tub^ compared to control are Lrg1 (leucine rich alpha-2-glycoprotein 1), Serpinf1 (serpin family F member 1), Sdc2 (syndecan 2), Marcks (myristoylated alanine rich protein kinase C substrate) and Ramp1 (receptor activity modifying protein 1), where Serpinf1 is an inhibitor of angiogenesis and the other mRNA induce angiogenesis, angiogenic sprouting and endothelial cell proliferation. Taking a closer look, the reduced mRNA levels in IKKβ^∆Tub^ are rather normalized to baseline and the control mice at 14 days after I/R injury are increased, suggesting that at 14 days after I/R injury IKKβ^∆Tub^ present a normalized angiogenesis whereas the control mice at that time point start to display an increased angiogenesis.

### 2.4. Tubular Deletion of IKKβ Affect Glucose Metabolism after I/R Injury

**Figure 4:**
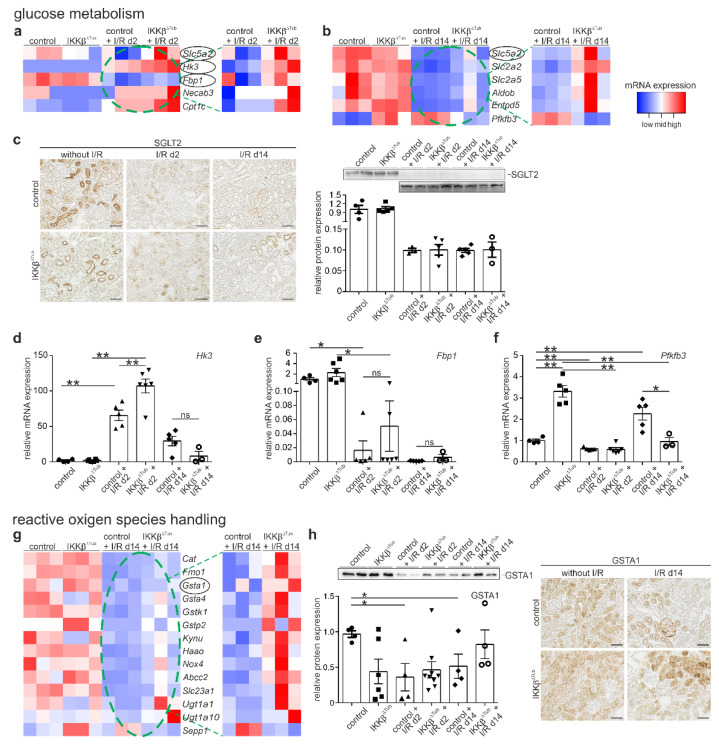
Tubular deletion of IKKβ affects glucose metabolism and reactive oxygen species handling after I/R injury. (**a**,**b**) heatmaps of significantly altered mRNA of proteins known to affect glucose metabolism at 2 days (**a**) and at 14 days (**b**) after I/R injury compared to baseline. Filter criteria were DESeq *p*-values < 0.05; fold change > 1.5. mRNA further analyzed are encircled. All heatmaps (**a**,**b**,**g**) show samples organized in columns and genes in rows, expression intensities are color-coded. The green stippled circle indicates mRNA of I/R groups used for relative expression in heatmaps by comparing only control with IKKβ^∆Tub^ at 2 days (**a**) and 14 days (**b**) after I/R injury. (**c**) Representative images of SGLT2 stained kidney sections and Western blot analysis from control and IKKβ^∆Tub^ at baseline, 2 days and 14 days after I/R injury. Magnification scale bar = 50 µm. Densitometrical evaluation is presented in % arithmetic means ± SEM of *n* = 3–5 per group. (**d**,**f**) real-time PCR of Hk3 (**d**), Fbp1 (**e**) and Pfkfb3 (**f**) mRNA expression of kidneys from control and IKKβ^∆Tub^ at baseline, 2 days and 14 days after I/R injury. Arithmetic means ± SEM of *n* = 3–6 per group. (**g**) heatmaps of significantly altered mRNA from proteins known to improve detoxification and reactive oxygen species handling at 14 days after I/R injury compared to baseline. Filter criteria were DESeq *p*-values < 0.05; fold change > 1.5. The green stippled circle indicates mRNA of I/R group used for relative expression in heatmaps by comparing only control with IKKβ^∆Tub^ at 14 days after I/R injury; (**h**) representative images of GSTA1 stained kidney sections and Western blot analysis from control and IKKβ^∆Tub^ at baseline, 2 days and 14 days after I/R injury. Magnification scale bar = 50 µm. Densitometrical evaluation is presented in % arithmetic means ± SEM of *n* = 4–9 per group. For (**c**–**f**,**h**) * *p* < 0.05, ** *p* < 0.01 using Mann–Whitney–U test (if *n* < 4 Lord test).

IKKβ is a kinase known to affect glucose metabolism, and it was shown further that glucose metabolism affects the degree of injury and the capacity of tissue regeneration. Therefore, we analyzed the mRNA levels of genes important in glycose metabolism and energy expenditure from the RNASeq data. Significantly higher mRNA levels were found for Slc5a2 (sodium glucose transporter 2, SGLT2), Hk3 (hexokinase 3, HK3), Fbp1 (fructose-1,6.bisphosphatase, FBP1), Necab3 (N-terminal EF-hand calcium binding protein 3, NECAB3), and Cpt1c (carnitine palmitoyltransferase 1C, CPT1C), see (Figure 4a). SGLT2 is the main transporter for proximal tubular glucose uptake, NECAB3 and HK3 are an inducer and an enzyme of glycolysis, CPT1C is a known nutrient sensor in metabolic stress conditions, and FBP1 is an important enzyme in gluconeogenesis. At 14 days after I/R injury, the metabolic enzyme normalizes to a base line level for IKKβ^∆Tub^, whereas they remain low in control mice (Figure 4b). To investigate glucose homeostasis, we measured renal SGLT2 expression levels by performing Western blot and immunohistochemical analysis of SGLT2. At baseline, no difference between IKK^β∆Tub^ and control was observed. At 2 and 14 days after I/R injury, a strong reduction in SGLT2 expression was encountered with no difference between IKKβ^∆Tub^ and control (Figure 4c). In addition, we analyzed mRNA expression of Hk3, Fbp1 and Pfkfb3. Hk3 mRNA was significantly higher at 2 days after I/R injury in both groups with strongest induction in IKKβ^∆Tub^ and control; at 14 days after I/R injury, Hk3 mRNA expression normalized to a base line level in both groups (Figure 4d). Fbp1 mRNA was not significantly different between strains at baseline; at 2 days after I/R injury, Fbp1 mRNA was strongly reduced in both groups, with significantly higher expression in IKKβ^∆Tub^ compared to control (Figure 4e). At 14 days after I/R injury, Fbp1 mRNA remained lower as a baseline level and was increased more in IKKβ^∆Tub^ compared to control. Pfkfb3 mRNA was significantly augmented upon tubular *IKKβ* deletion at baseline (Figure 4f). At 2 days after I/R injury, the high Pfkfb3 mRNA expression in IKKβ^∆Tub^ compared to control was abolished and was even inversed at 14 days after I/R injury where Pfkfb3 mRNA was significantly higher in control compared to IKKβ^∆Tub^. Results confirm RNA sequencing data.

### 2.5. Tubular Deletion of IKKβ Improves Detoxification and Reactive Oxygen Species Handling after I/R Injury

Reentry of oxygenated blood into ischemic tissue results in production of reactive oxygen species (ROS) with induction of cell dysfunction. The cellular response to ROS defense might be one of mechanisms for how *IKKβ* deletion reduced I/R injury. Therefore, we analyzed changes of mRNA related to ROS handling and its defense. At 2 days after I/R injury, only minor alterations were found. At 14 days after I/R injury, significantly higher mRNA levels were found for Cat (catalase), Gsta1, Gsta4, Gstk1 and Gstp2 (glutathione S-transferase alpha 1, and 4, kappa 1 and pi 2), and Haao (3-hydroxyanthranilate 3,4-dioxygenase), Kynu (kynureninase) and Slc23a1 (ascorbic acid transporter) in IKKβ^∆Tub^ compared to control. In general, IKKβ^∆Tub^ demonstrated mRNA levels which were rather normalized to the baseline level in comparison to control presenting significantly lower mRNA expression of genes for reactive oxygen handling (Figure 4g). In addition, we performed Western blot and immunohistochemical analysis of GSTA1. Significantly lower expression for GSTA1 was found in control at 2 and 14 days after I/R injury compared to IKKβ^∆Tub^ and to control at baseline and normal values were found for IKKβ^∆Tub^ at 14 days after I/R injury (Figure 4h).

### 2.6. Tubular Deletion of IKKβ Improves Proliferation, Tissue Regeneration and Reduces Fibrosis

**Figure 5:**
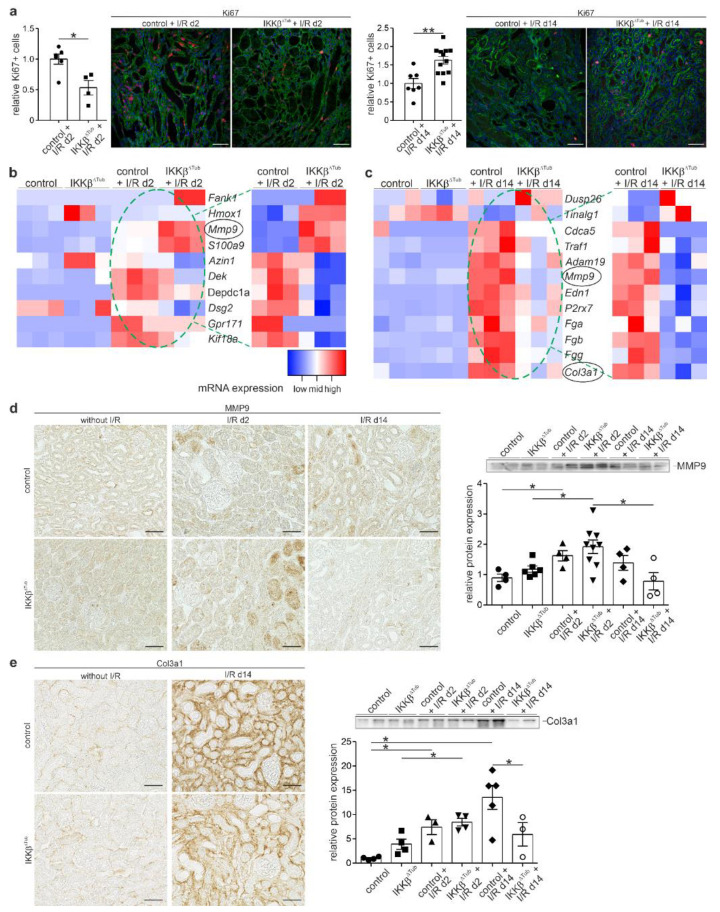
Tubular deletion of IKKβ affects proliferation, tissue regeneration and fibrosis. (**a**) assessment of Ki67-positive proliferating cells in control and IKKβ^∆Tub^ at 2 days and 14 days after I/R injury showing the semi-quantitative evaluation and representative images. Scale bar = 50 µm. Arithmetic means ± SEM of *n* = 4–12 per group; (**b**,**c**) heatmaps of significantly altered mRNA for cell proliferation, apoptosis and fibrosis at 2 days (**b**) and at 14 days (**c**) after I/R injury. Filter criteria were DESeq *p*-values < 0.05; fold change > 1.5. All heatmaps show samples organized in columns and genes in rows, expression intensities are color-coded. (**d**,**e**) assessment of MMP9 (**d**) and Col3a1 (**e**) showing densitometric analysis of respective Western blots and representing images for each strain and time point. Scale bar = 50 µm. Arithmetic means ± SEM of *n* = 3–9 per group. The green stippled circle indicates mRNA of I/R groups used for relative expression in heatmaps by comparing only control with IKKβ^∆Tub^ at 2 days (**c**) and 14 days (**d**) after I/R injury. For (**a**,**d**,**e**) * *p* < 0.05, ** *p* < 0.01, Mann–Whitney–U test (if *n* < 4 Lord test).

Because we have observed an ameliorated kidney function and morphology in IKKβ^∆Tub^ in comparison to control at 14 days after I/R injury, we examined the RNA sequencing for mRNA inducing cell proliferation, tissue regeneration and affecting fibrosis. In IKKβ^∆Tub^ in comparison to control at 2 days after I/R injury, we identified among the significantly upregulated mRNA factors which are anti-apoptotic such as Fank1 (fibronectin type 3 and ankyrin repeat domains protein 1), Hmox1 (heme oxygenase 1), and factors inducing tubular repair and recovery such as Mmp9 (MMP9) and S100a9 (calcium-binding protein complex S100A9) (Figure 5a). Interestingly, in IKKβ^∆Tub^ compared to control at 2 days after I/R injury, among the significantly reduced mRNA, we found genes promoting cell proliferation such as Azin1 (antizyme inhibitor 1), Depcd1a (DEP domain containing 1), Dsg2 (Desmoglein2), Gpr171 (G protein-coupled receptor 171), and Kif18a (kinesin family member 18A).

On the contrary to 2 days after I/R injury, in IKKβ^∆Tub^ compared to control at 14 days after I/R, we found significantly increased mRNA where its protein is known to induce cell proliferation such as Tinagl1 (tubulointerstitial nephritis antigen like 1) and Dusp26 (dual specificity phosphatase 26) shown to reduce ROS formation and fibrosis (Figure 5b). At 14 days after I/R injury, among the significantly downregulated mRNAs in IKKβ^∆Tub^ compared to control, we identified Cdca5 (cell division cycle associated 5) suspending cell proliferation, Traf1 (TNF receptor associated factor 1) promoting inflammation, and many pro-fibrotic factors such as Adam19 (ADAM metallopeptidase domain 19), Mmp9 (matrix metalloprotease 9), Edn1 (endothelin-1), P2rx7 (purinergic receptor P2X7), Fga, Fgb and Fgg (fibrinogen alpha, beta and gamma chain), and Col3a1 (collagen III alpha 1).

To verify RNA sequencing results, we assessed the proliferating Ki67-positive epithelial cells of IKKβ^∆Tub^ compared to control at 2 and 14 days after I/R injury (Figure 5c). In correlation with mRNA altered, we found at 2 days after I/R injury a reduced number of proliferating cells in IKKβ^∆Tub^ compared to control. At 14 days after I/R injury, however, we found a significantly increased number of Ki67-positive cells in IKKβ^∆Tub^ compared to control. Next, we determined MMP9 expression by Western blot analysis and immunohistochemistry, showing an increased expression level at day 2 after I/R injury and a reduced expression level at 14 days after I/R injury in IKKβ^∆Tub^ compared to control (Figure 5d). Finally, we also determined collagen III alpha1 expression by Western blot and immunohistochemical analysis showing strong interstitial expression at 14 days after I/R injury in control and significantly less in IKKβ^∆Tub^, which was confirmed by Western blot (Figure 5e).

## 3. Discussion

We demonstrated that tubular deletion of IKKβ ameliorated renal function parameter and kidney morphology at 2 days and long term at 14 days after I/R injury. Furthermore, mRNA signature of IKKβ^∆Tub^ at 14 days after I/R injury was rather adapted to control at baseline compared to control at 14 days after I/R injury. Besides its role in inhibiting inflammatory NF-ĸB signaling, it was shown recently to have other substrates as well, including enzymes for metabolism [10]. Deregulated glucose metabolism was implicated in mortality rate and degree of acute kidney injury [11]. Already at baseline, tubular *IKKβ* deletion demonstrated higher mRNA expression levels of signaling and transport proteins, for enzymes and mitochondrial uncoupling. PFKFB3 was shown earlier to be phosphorylated by IKKβ leading to its inhibition and concomitant reduction in aerobic glycolysis [12]. This complies with *IKKβ* deletion induced a higher Pfkfb3 mRNA expression level. Furthermore, other mRNA for glycolytic enzymes such as Hk2 and Ldhd were elevated in IKKβ^∆Tub^. Interestingly, we found many mRNAs of proteins for mitochondrial uncoupling to be higher in IKKβ^∆Tub^, suggesting that cellular *IKKβ* deletion promotes the Warburg effect with augmented glycolysis and mitochondrial uncoupling. In addition, significantly higher levels of mRNA important for fatty acid uptake and biosynthesis were identified. Fatty acids are the preferred fuel of proximal tubule cells and may be used to cope with the Warburg effect. Furthermore, among the altered mRNA level in IKKβ^∆Tub^ found protective in I/R injury most significantly higher was *Grem1* (Gremlin1), and most significantly lower were *Tbxa2r*, *Mtfp1* (MTFP1), and *Gm853*. Gremlin1 was shown to protect renal epithelial cells for I/R injury induced apoptosis [13]. Polymorphisms in Tbxa2r were associated with higher blood pressure [14], platelet aggregation and ischemic stroke [15]. MTFP1 is a key protein for mitochondrial fission; its reduced expression would inhibit fission, promoting mitochondrial inner membrane fusion and thereby contribute to the reduction in apoptosis induced by I/R injury [16]. Gm853 is coding for leucine decarboxylase 1 where its role remains elusive.

### 3.1. Tubular IKKβ Deletion Induced Angiogenesis

Microvascular dysfunction is central to the severity of acute kidney injury [9]. After I/R injury, capillary rarefaction in the recovery phase is followed by tubulointerstitial fibrosis and might also be a key-player in the pathophysiology of AKI-to-CKD transition [17]. Tubular *IKKβ* deletion prevented capillary rarefaction at 2 days and in the recovered kidney at 14 days after I/R injury. In agreement, RNA sequencing analysis revealed higher mRNA levels of pro-angiogenic factors and reduced levels of angiogenesis inhibitors in IKKβ^∆Tub^ compared to control at 2 days after I/R injury and compared to both strains at baseline. At 14 days after I/R, however, angiogenic factors are normalized to baseline in IKKβ^∆Tub^, whereas control at 14 days after I/R started to display ongoing angiogenesis by expressing pro- and anti-angiogenic factors. These results show in general that control mice after I/R injury demonstrated higher microvascular dysfunction, which was preserved upon tubular *IKKβ* deletion.

### 3.2. Tubular IKKβ Deletion Improves Glucose Metabolism

The kidney consumes a significant amount of energy to support active transport of molecules and excretion functions. At 2 days after I/R injury, we found strongly reduced SGLT2 levels important for glucose uptake which did not differ between strains and remained low at 14 days after I/R injury. At 2 days after I/R injury, mRNA for glucose metabolism such as Hk3 (glycolytic enzyme) and Fbp1 (enzyme for gluconeogenesis) were found to be significantly higher upon tubular *IKKβ* deletion. Crucial intermediates of the glycolytic pathway were demonstrated to ameliorate the severity of renal damage upon I/R injury by either administration prior or during the post-ischemic reperfusion period [18]. For fructose 1,6-diphosphate it was shown to attenuate renal cell injury. In addition to glycolysis, gluconeogenesis was shown to play a pivotal role in acute kidney injury by reducing patient mortality [11,19]. The kidney uses mainly lactate as a substrate for gluconeogenesis particularly during fasting and stress conditions. In our analysis, we observed increased expression of Fbp1 mRNA at 2 days after I/R injury compared to control, an important enzyme for glyconeogenesis. Both, HK3 and FBP1 were found to be expressed in the proximal tubule; however, they were most probably not in the same cells and may be due to the well-known cell heterogeneity of the renal proximal tubule [20]. At 14 days after I/R injury, in IKKβ^∆Tub^, mRNA levels of proteins involved in the glucose metabolism normalize to baseline levels, whereas they remain low in control mice, suggesting that tubular *IKKβ* deletion ameliorates glucose handling in favor of tissue and body recovery. Surprisingly, the IKKβ substrate Pfkfb3 remained unaltered between strains at day 2 after I/R injury and increased only in control at 14 days after I/R injury. We do not have an explanation for this, but this may be an aberrant glycolysis and re-programming of glucose metabolism in the long term after I/R injury.

### 3.3. Tubular IKKβ Deletion Improves Kidney Antioxidant Defense

At 2 days after I/R injury, we detected only minor changes of mRNA related to ROS, assuming that at this time point no regulation of ROS altering genes occurs on the mRNA level. At 14 days after I/R injury compared to control and rather towards the baseline level, IKKβ^∆Tub^ demonstrated higher mRNA levels of anti-oxidative stress enzymes, several glutathione-S-transferase subunits, enzymes involved in NAD+ synthesis and vitamin C transporter to inhibit late ROS accumulation. The relative higher levels of anti-oxidative stress enzymes in IKKβ^∆Tub^ at 14 days after I/R injury may be ascribed to the improved overall outcome, since its baseline expressions remained unaltered and were not affected by the tubular *IKKβ* deletion.

Tubular *IKKβ* deletion reduces apoptosis in the short term and increases proliferation in the long term with concomitant tissue regeneration and reduced fibrosis. Genes-expression analysis demonstrates that at 2 days after I/R injury, in IKKβ^∆Tub^, higher mRNA levels for anti-apoptotic protein Fank1 [21] and especially for heme oxygenase 1, which was shown to prevent apoptosis, to promote cell survival, circulatory integrity, and immunomodulation [22]. In addition, IKKβ^∆Tub^ showed higher mRNA levels in factors inducing tubular repair and recovery such as Mmp9 and S100a9. MMP9 was differentially expressed at 2 days after I/R injury with higher Mmp9 mRNA levels in IKKβ^∆Tub^ compared to control and at 14 days after I/R injury with lower Mmp9 mRNA values in IKKβ^∆Tub^ compared to control. This differential expression pattern displays the different function. MMP9 can attenuate apoptosis via soluble SCF-c-kit pathway 24 hours after I/R injury, which is in agreement with reduced histologic lesions and kidney function [23]. However, at late stages in acute kidney injury (14 days after I/R injury), MMP9 may have a pathogenic role in AKI-CKD transition. In addition, at that late time point, MMP9 contributes to macrophage recruitment and promotes thereby epithelial-mesenchymal-transition and fibrosis. Thus, in IKKβ^∆Tub^ compared to control, MMP9 expression is altered in favor of preventing renal damage and promoting tissue regeneration. At 2 days after I/R injury, mRNA inducing cell proliferation, such as Azin1 [24], Depcd1a [25], Dsg2 [26], Gpr171 [27] and Kif18a [28], were lower in IKKβ^∆Tub^ compared to control, which is in line with the evaluation of Ki-67 positive cells at 2 days after I/R injury. This phenomenon is inversed at 14 days after I/R injury with higher proliferation rates in IKKβ^∆Tub^ compared to control and together with higher mRNA level of Tinagl1 [29] inducing cell proliferation and lower levels of Cdca5 [30] suspending cell proliferation. These time differences in cell proliferation are in line with transient cell cycle arrest, which was shown to be protective in acute kidney injury [31]. Furthermore, in agreement with the observation of diminished fibrosis and collagen 3 expression, we observed higher Dusp26 mRNA level at 14 days after I/R injury in IKKβ^∆Tub^ compared to control, which was presented earlier to reduce ROS formation and fibrosis [32]. In addition, mRNA of fibrosis promoting factors such as Adam19 [23], P2rx7 [33] and fibrinogen subunits Fga, Fgb and Fgg [34,35] were significantly reduced in IKKβ^∆Tub^ compared to control at 14 days after I/R injury supporting the important role of IKKβ in ameliorating the renal outcome in the long-term after I/R injury.

In summary, following I/R kidney injury, tubular deletion of *IKKβ* improves kidney function, renal morphology and supports tissue regeneration as well as reduces fibrosis. This effect was associated with angiogenesis, and with a gene–expression signature indicating improved glucose metabolism, antioxidant defense, and reduced apoptosis in the early phase followed by increased proliferation during the late regeneration phase.

From our data, we can conclude that prevention of tubular NF-ĸB-mediated response does not solely reduce inflammatory cytokine and chemokine production; beyond that, it affects so far unknown targets such as angiogenesis, metabolism and ROS handling. It thereby improves kidney function early after I/R injury and in the recovery phase for the overall ameliorated long-term outcome. Pharmacological intervention targeting NF-ĸB pathway after I/R injury might be a possible medical strategy. Other mechanisms accounting for the alteration of cellular metabolism and stress handling after I/R injury should be further investigated.

## 4. Materials and Methods

### 4.1. Animal Experimentation

All mice experiments were conducted according to the National Institute of Health guide for the care and usage of laboratory animals and the Swiss law for the welfare of animals. All experiments were approved by the Cantonal Veterinary Office (Canton of Fribourg, Switzerland, FR27451, 2016_04_FR). All protocols were reviewed by the University’s Animal Welfare and Ethics Review Board before experimentation. Mice were housed in a SPF facility in ventilated cages under conditions of stable temperature (23 °C) and humidity with free access to chow and tap water in a 12-h day/night cycle. Breeding and genotyping were performed according to standard procedures. IKKβ^fl/fl^ mice [36] have been and were crossed with Pax8-rt-TA-LC1^Cre^ mice [37]. For the induction of tubular knockout of IKKβ, 6 weeks old IKKβ^fl/fl^ (termed control) and Pax8-rt-TA-LC1^Cre^/IKKβ^fl/fl^ (termed IKKβ^∆Tub^) received 0.2 mg/mL doxycycline (Sigma Aldrich, Taufkirchen, Germany) in the drinking water for two weeks followed by one week wash out. Mice were anaesthetized with a combination of ketamine (65 mg/kg), xylazine (13 mg/kg) and azepromazine (2 mg/kg). Analgesia was performed pre- and postoperatively using buprenorphine (0.01 mg/kg). Flank incision was performed on heating pads, and a vascular clamp was applied for 30 min on the left renal pedicle. Reperfusion was monitored visually, and mice were closed. After surgery, mice were monitored twice daily. All analyses were carried out by investigators who were blinded to the experimental conditions.

### 4.2. Blood and Urine Collection/Analysis

In addition, 24 h and 13 days after IRI, urine were collected for 24 h, and mice were anaesthetized and blood samples were drawn and centrifuged at 1000× *g* for 5 min at 4 °C to analyze renal function parameters. Creatinine concentrations were enzymatically determined in urine and plasma samples using LT-SYS CREATININ PAP kit (Labor + Technik Eberhard Lehmann GmbH, Berlin, Germany). LT-SYS Harnstoff was used to determine urea concentration in blood serum (Labor + Technik Eberhard Lehmann GmbH, Berlin, Germany). Protocols of creatinine and urea kit were adjusted to lower volumes, and absorption was measured using the microplate reader (Tecan Infinite 200 Pro, Tecan, Switzerland, Tecan i-control). Serum and urine electrolytes were quantified with flame-photometry (EFOX 5053, Eppendorf). Proteinuria was assessed using Bio-Rad Protein Assay (Bio-Rad) according to the manufacturer’s instructions.

### 4.3. Fixation and Tissue Processing for Immunohistochemistry and Immunoblotting

In addition, 48 h and 14 days after IRI, mice were anesthetized by intraperitoneal injection of ketamine/xylazine, and kidneys were removed and shock-frozen for biochemical evaluation or perfused retrogradely through the aorta by using 3% PFA in Caco-Sucrose buffer containing 93 mM cacodylate acid, 60 mM sucrose, 6.67% Haes, 3.25 mM magnesium chloride hexahydrate. Perfusion-fixed specimens were post-processed for cryo-, paraffin-, and epon-embedding for further histochemical, light and electron microscopy analysis. For immunoblotting, total and membrane fractions were prepared from shock-frozen renal cortices. For total fraction RIPA buffer (20 mM Tris-Hcl, 150 mM NaCl, 1 mM EDTA, 1% Nonidet P-40, 0,25% sodium deoxycholate) containing protease inhibitors and phosphatase inhibitors (Roche) was used for extraction, and membrane fractions were prepared using a 0.25 mol/L sucrose buffer containing triethanolamine (0.13%), protease inhibitors and phosphatase inhibitors (Roche) and processed as described [38]. Protein content was determined using the Pierce™ BCA Protein Assay Kit (Thermo Fisher Scientific, Waltham, MA, USA).

### 4.4. RNA Isolation, Reverse Transcription, Real-Time PCR and RNA Sequencing

RNA was isolated from frozen kidney tissue using NucleoSpin RNA columns (Macherey-Nagel, Düren, Germany). Residual DNA was digested using DNaseI Kit (AMPD1, Merck, Darmstadt, Germany). In addition, *n* = 2 RNA samples per group were pooled for RNA sequencing. Furthermore, 2 µg RNA were reverse transcribed into cDNA in the presence of M-MLV reverse transcriptase (Promega), dNTP and random hexamer primer. For quantitative real-time PCR, cDNA was added to TaqMan Fast Advanced master mix (Applied Biosystem, 4444556), TATA-box binding protein (Tbp; Mm00446973_m1, Thermo Fisher Scientific, Germany), hexokinase 2 (Hk2; Mm00443385_m1), hexokinase 3 (Hk3; Mm01341942_m1), Fructose-1,6-Bisphosphatase 1 (Fbp1; Mm00490181_m1) or 6-phosphofructo-2-kinase (Pfkfb3; Mm00504650_m1) and subjected to qPCR.

### 4.5. RNA-Seq, Sequence Details

Paired-end sequencing libraries were constructed for replicates of each genotype from the total RNA isolated, employing the True Seq stranded mRNA Poly ATrue Seq stranded mRNA Poly A library kit. Subsequent sequencing was performed on the Illumina NovaSeq_6000 (2 × 50 bp) using standard protocols.

RNA-Seq pipeline. We used our in-house RNA-Seq pipeline to map and align the sequenced data (https://github.com/nf-core/rnaseq (accessed on 9 November 2021). The workflow processed the raw data from the sequencer with FastQC v0.11 [39], and Trimgalore v0.4. [40] aligned the reads with STAR v2.5.2b [41] and generated gene counts with featurecounts v1.5.2 [42] and StringTie v1.3.3b [43]. Quality control was assessed throughout with RSeqQC [44] dupRadar [45], Preseq [46] and MultiQC v1.4 [47]. As reference genomes, we used GRCm38 Mus musculus genome (Genome Reference Consortium Mouse Build 38 and GenBank Assembly ID: GCA_000001635.8), with the total of reads per sample aligning on average 83.56%.

RNA-Seq analysis. Differentially expressed genes were identified by comparing expression profiles of the different genotypes. Statistical analysis preformed using R v3.6.3. To identify differentially expressed genes between groups and time points, we used DESeq2 R package v1.26.0 [48]. DESeq2 was used for executing pairwise comparisons between genotypes. This statistical tool is based on a negative binomial distribution model with dispersion trend smoothing and was also used to determine the normalized reads counts per sample by estimating size factors to control for library size, followed by a log2 transformation of the raw count data using DESeq2. Raw data and processed data from RNA sequencing analysis were uploaded on GEO (https://www.ncbi.nlm.nih.gov/geo/ (accessed on 10 November 2021); accession number: GSE207430).

### 4.6. RNA In Situ Hybridisation

To detect Ikkβ mRNA derived from exon 3, in situ hybridisation was performed on 5 µm paraffin sections with BaseScope Detection Reagent kit v2-RED (cat.no. 323910, Bio-Techne, Abingdon, UK) and a 3-ZZ probe against mouse Ikkβ mRNA directed against exon 3 (238–332 bp coding sequence, cat.no. 541151). BaseScope was carried out according to manufacturer’s instructions. To verify mRNA quality, a probe against the housekeeping gene peptidylprolyl isomerase B (cat.no. 701071, accession no. NM_011149.2) served as positive control. As negative control served a probe against the bacterial gene DapB (cat.no. 701011, accession no. EF191515). Sections were counterstained with DAPI (4′,6-Diamidin-2-phenylindol). Z-stack images were taken using Leica Axiovert microscope and Fiji ImageJ was used to determine the percentage of Ikkβ-positive/devoid tubular epithelial cells.

### 4.7. SDS-PAGE and Immunoblotting

Proteins were solubilized, and SDS gel electrophoresis was performed on 10–15% polyacrylamide gels. After electrophoretic transfer of the proteins to nitrocellulose membranes, equity in protein loading and blotting were verified by membrane staining using 0.1% Ponceau red. Membranes were probed with primary antibodies and then exposed to HRP-conjugated secondary antibodies (Dianova, Hamburg, Germany). Immunoreactive bands were detected by chemiluminescence using Immobilon Western HRP substrate (Millipore, Darmstadt, Germany) in combination with the chemiluminescence imaging system Fusion SL (Peqlab, Erlangen, Germany) and further analyzed using ImageJ software. Resulting values are presented in percent of control values obtained from the control group.

### 4.8. Immunohistochemistry

In addition, 5 µm Paraffin sections were antigen-retrieved by high-pressure cooking, followed by blocking endogenous peroxidase with 3% H_2_O_2_ in 100% methanol and by a blocking step using 5% skim milk/PBS. Sections were incubated with the primary antibody directed against Col3a1, SGLT2, TOM20, GSTA1 or MMP9 overnight followed by the suitable HRP-coupled secondary antibody (Dianova). Signals were generated using 3,3′-diaminobenzidine. Images were captured using Keyence BZ-X (software BZ-X800 Viewer; Keyence, Neu-Isenburg, Germany).

### 4.9. Cellular Proliferation

Cell proliferation was assessed using antigen-retrieved paraffin sections co-stained with an antibody against the proliferation marker Ki-67 and DAPI. Images were captured using Abberior Facility Line (Abberior Instruments GmbH, Göttingen, Germany). Channels were merged, and signal was evaluated using Fiji/ImageJ software. The number of Ki-67–positive epithelial nuclei per visual field (40× magnification) [49,50] were counted. About ten randomly chosen areas in renal cortex were analyzed.

### 4.10. Antibodies

The following antibodies were used: rabbit anti-MMP9 (Proteintech, cat.no.: 10375-2-AP); rabbit anti-Ki67 (Thermo Fisher Scientific Invitrogen, MA5-14520); rabbit anti-Gsta1 (Proteintech, cat.no.: 14475-1-AP); rabbit anti-Col3a1 (Proteintech, cat.no.: 22734-1-AP); rabbit anti-Tom20 (Proteintech, cat.no.: 11802-1-AP) and rabbit anti-SGLT2 (gift. Of H. Koepsell).

### 4.11. Histology and Tubular Injury Score

In addition, 2 µm paraffin tissue sections were stained for Masson trichrome using standard protocol. Images were captured using Keyence BZ-x800e microscope (Keyence Deutschland GmbH, Neu-Isenburg, Germany) at 20× magnification. The tubular injury was assessed according to the scoring system blinded to the genotype of mice as published earlier [51]. The evaluation accounts cortical tubules with epithelial necrosis, loss of the brush border, and tubular dilation. A five-point scale was used: 0, normal kidney; 1, 1–25% of tubular injury; 2, 24–50% of tubular injury; 3, 50–75% of tubular injury; 4, 75–100% of tubular injury.

### 4.12. Electron Microscopy and Morphometry

For electron microscopy, kidney specimens were post-fixed overnight in 1.5% paraformaldehyde, 1.5% glutaraldehyde and 0.05% picric acid in cacodylate buffer, incubated with 1% osmium tetroxide and embedded in Epon. Semi-thin sections were cut and stained with 0.1% toluidine blue using standard protocol. Kidney cortices and outer medulla were imaged and captured by Keyence BZ-x800e microscope, and the tubulointerstitial capillary density was determined by estimating the fraction of the tubular circumference that is in close contact with capillaries. Approximately 50 cortical tubules per animal were evaluated stereologically, and capillary-epithelial contact areas were calculated as performed previously [52].

Ultrathin sections were cut using an ultramicrotome (Leica EM UC7) and stained with uranyl acetate and lead citrate. Ultra-thin sections were examined using TEM (JEM 1400 plus, JOEL), and images were captured with TemCam F416 (TVIPS).

### 4.13. Statistical Data Analysis

Statistical comparisons were done with the GraphPad Prism Software Package 5 (GraphPad Software, La Jolla, CA, USA) using the Mann–Whitney–U test or Lord test [53]. *p*-values of <0.05 were judged to be statistically significant. Asterisks are used in the figures to explicitly demonstrate the statistical significance (* *p*  <  0.05; ** *p*  <  0.01; *** *p*  <  0.001).

## Data Availability

The data are contained within the article or supplementary material. Raw data and processed data from RNA sequencing analysis are uploaded on GEO (https://www.ncbi.nlm.nih.gov/geo/ (accessed on 10 November 2021); accession number: GSE207430).

## Supplementary Materials

The supporting information can be downloaded at: www.mdpi.com/article/10.3390/ijms231710199/s1.
